# Advancements in precision medicine: multi-omics approach for tailored metformin treatment in type 2 diabetes

**DOI:** 10.3389/fphar.2024.1506767

**Published:** 2024-11-28

**Authors:** Najeha Rizwana Anwardeen, Khaled Naja, Mohamed A. Elrayess

**Affiliations:** ^1^ Biomedical Research Center, Qatar University, Doha, Qatar; ^2^ College of Medicine, QU Health, Qatar University, Doha, Qatar

**Keywords:** metformin, genomics, metabolomics, microbiomics, precision medicine

## Abstract

Metformin has become the frontline treatment in addressing the significant global health challenge of type 2 diabetes due to its proven effectiveness in lowering blood glucose levels. However, the reality is that many patients struggle to achieve their glycemic targets with the medication and the cause behind this variability has not been investigated thoroughly. While genetic factors account for only about a third of this response variability, the potential influence of metabolomics and the gut microbiome on drug efficacy opens new avenues for investigation. This review explores the different molecular signatures to uncover how the complex interplay between genetics, metabolic profiles, and gut microbiota can shape individual responses to metformin. By highlighting the insights from recent studies and identifying knowledge gaps regarding metformin-microbiota interplay, we aim to highlight the path toward more personalized and effective diabetes management strategies and moving beyond the one-size-fits-all approach.

## Introduction

Type 2 Diabetes (T2D) is a complex and chronic metabolic disorder that has become a major global health issue. The impact of this disease is significant, exerting substantial pressure on healthcare systems and affecting a considerable portion of the population (Pasquel et al., 2021). T2D is characterized by high blood glucose levels due to inadequate production of insulin or impaired insulin function, including a less drastic and more progressive loss of β-cell secretory capacity. It can cause major complications, impacting several organ systems and reducing the quality of life for those affected with the condition (Yun and Ko, 2021). As we explore the complex nature of T2D and its widespread prevalence, the healthcare sector is perceiving the rise of precision medicine as a hopeful direction. Precision medicine is an emerging concept in clinical research that concentrates on treating diseases by collecting data from various sources to make patient-specific decisions. It represents categorising people into subpopulations and identifying specific treatment options that benefit those populations (Woodcock, 2007). Personalised medicine involves focusing on individualized details such as clinical diagnosis, laboratory assessments, imaging, including external factors such as environmental, demographics and lifestyle (Oh et al., 2022).

T2D patients showcase heterogeneity in the phenotype of the disease and as well in treatment response and disease progression. While non-adherence to treatment explains much of the difference, there might be a possibility that it stems from diverse physiological factors among patients. Some individuals may respond well to one drug but not to another due to difference in the underlying cause of their disease condition (Unnikrishnan et al., 2021). The tolerance to side effects of these antidiabetic medications also differs significantly between individuals. In addition, glycemic control in diabetic individuals involves not just exogenous insulin, but also range of pharmacologic treatments offering effects such as enhancing insulin sensitivity, stimulating insulin release, and slowing down the adsorption of glucose in the intestines (Malandrino and Smith, 2011). In this context, metformin, a well-established initial treatment (Maruthur et al., 2016), distinguishes itself for its hypoglycemic ability and its broader effects such as enhanced endothelial function, reducing oxidative stress and insulin resistance, improving lipid profiles and managing fat redistribution (Rojas and Gomes, 2013).

Metformin, a biguanide agent, is the preferred first line oral medication for the treatment of hyperglycemia in type 2 diabetes. Metformin is created synthetically, comprising of two coupled molecules of guanidine (biguanide) (Wilcock and Bailey, 1994). It is hydrophilic with a pKa value of 11.5 and predominantly remains as an organic cation at physiological pH (Pentikäinen et al., 1979). Metformin is primarily absorbed in the upper small intestine and exhibits variable pharmacokinetics, and its oral bioavailability (F%) is limited (Pentikäinen et al., 1979; Graham et al., 2011). Metformin’s brief plasma half-life of 2–6 h results in a steady-state metformin plasma concentration of approximately 4–15 μM (∼0.5–2.0 μg/mL) in individuals with T2D (Graham et al., 2011). It reduces basal and postprandial glucose and increases glucose tolerance. The precise underlying mechanisms of metformin are still unknown; however, the reduction of glucose is due to decreased production in the liver due to the activation of AMP-activated protein kinase (AMPK). Contradicting evidence suggests that activation of AMPK has no direct impact on the glucose synthesis (Foretz et al., 2023a; Foretz et al., 2019), and the use of millimolar quantities of metformin in the studies (He and Wondisford, 2015; LaMoia and Shulman, 2021) has raised doubts about the physiological relevance of these processes. In fact, research has demonstrated that glucose synthesis in primary mouse hepatocytes is suppressed by relevant micromolar metformin doses through processes that are independent of alterations in adenine nucleotide levels (Foretz et al., 2023a; Alshawi and Agius, 2019; Ma et al., 2022). Additionally, metformin can also elevate skeletal muscle’s absorption of glucose, thereby increases insulin sensitivity. Metformin does not go through hepatic metabolism and is excreted unaltered in the urine (Guo and Priefer, 2021; Sansome et al., 2020). It is noteworthy that metformin can accumulate to higher amounts in a variety of tissues than in plasma. This underscores the inconstancy surrounding the appropriate concentrations of metformin for *in vitro* research (Wilcock and Bailey, 1994; Graham et al., 2011). Despite its commendable safety and effectiveness, there are notable differences in glycemic response in individuals with metformin therapy (DeFronzo and Goodman, 1995), probably due to uncertainty in the pharmacokinetics and pharmacodynamics of metformin.

Pharmacogenomics plays an important role in management of T2D by taking into account the genetic propensity and potential pharmacogenetic variations associated with antidiabetic agent like metformin (Avery et al., 2009). Research indicate that genetic variations account for 34% of metformin response variability, suggesting that genetic makeup of patients plays a major role in their reaction to treatment which may not be evident from their visible traits (Zhou et al., 2014). Similar to other complex traits, glycemic response to metformin depends on possible interplay between genetic and environmental factors. In addition, metformin also causes adverse side effects such as diarrhea and nausea which is experienced by 30% of patients, and some individuals experience, to a lesser extent, lactic acidosis. Even with metformin’s remarkable safeness and efficacy, over 38% of diabetic population still struggle to achieve glycemic targets with metformin. Regardless of the identification of gene variations that impact the pharmacokinetics or mechanism of action of metformin, these variants explain only a small portion of the diversity in metformin response (Song et al., 2008; Shu et al., 2007). Therefore, research is rapidly progressing beyond genomes in the era of personalized medicine to find clinically relevant molecular and metabolic indicators linked to a drug’s response.

**FIGURE 1:**
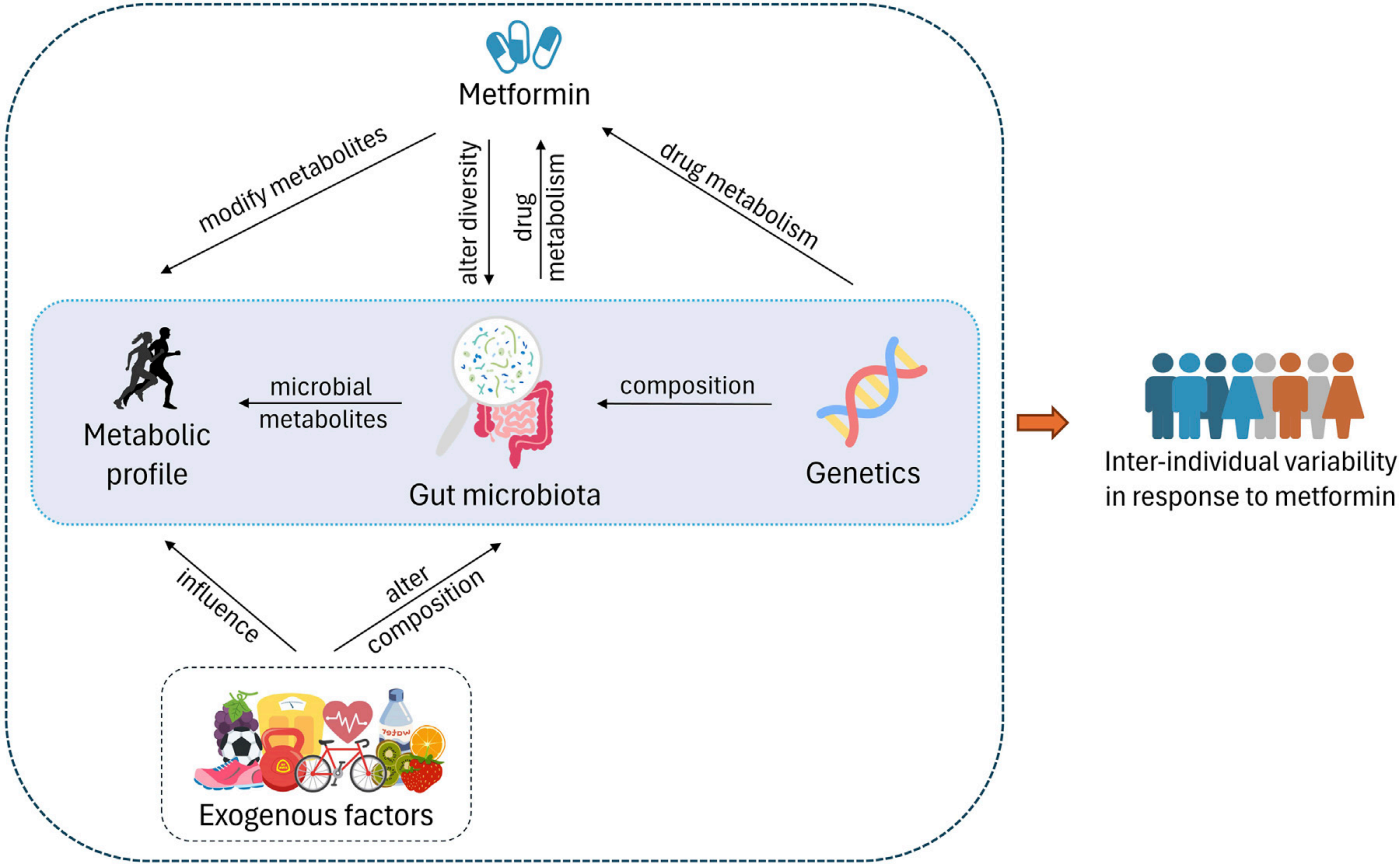
Host genetics play an important role in gut microbiota composition and also affect the drug metabolism. Exogenous factors such as exercise, diet, other lifestyle factors, and consumption of metformin alter the diversity of the microbiota. On the other hand, gut microbiota affects the drug metabolism via microbiota-related and -derived metabolites. These metabolites make up the metabolome as well. The interplay between these factors causes varied response to metformin in individuals.

In the current landscape of personalized medicine, the focus is shifting from solely examining genetic variations to a broader, more integrative approach. While genomics has provided valuable insights into how genetic differences can influence drug response (Jithesh et al., 2022), it has become evident that genetics alone cannot fully explain the variability in drug efficacy and safety across individuals (Zhou et al., 2022). Consequently, research is now extending into other ‘omics’ fields, such as metabolomics, microbiomics, and transcriptomics to uncover additional biomarkers that might better predict individual responses to therapies. For an optimum approach to personalized metformin treatment in diabetes, it is crucial to understand the effect of phenotypic variation in drug response and utilize the field of omics to enhance the ability to forecast outcome which will result in favourable modifications in patient care and preferred result (Pearson, 2016). Metabolomics has a significant potential to increase the knowledge of patient’s drug response as it provides information on distinct metabolic signatures associated with drug efficacy. Additionally, studies have demonstrated the role of metabolomics in exploring the pharmacodynamics of metformin in diabetic patients (Gong et al., 2012; Kim, 2021a). Environmental aspects such as lifestyle, medication, and the microbiome play an important role in the variability of drug response (Figure 1).

Increasing evidence suggests that microbiota, specifically gut microbiota contributes to the drug metabolism (Li and Jia, 2013). The complex interactions between the human body (host) and the gut microbiome require the development of innovative models to predict treatment outcomes accurately. (Wilson and Nicholson, 2017). Various research has concentrated on discovering the relationship between metabolic disorders and gut microbiome, although focus on diabetes has only just scratched the surface (Lee C. B. et al., 2021).

In this review, we explore the diverse factors contributing to the variability in metformin response among T2D individuals, with a particular focus on recent advancements in genomics, metabolomics, and microbiomics. While repurposing of metformin is being expanded to include a variety of pathophysiological disorders due to its pleiotropic modes of action (Foretz et al., 2023a), we aim to specifically examine the current knowledge on metformin-microbiota connection and its associated synergistic and/or detrimental effects in T2D individuals. Finally, we discuss the advantages of utilizing multi-omics approaches in order to emphasize the systemic changes induced by metformin treatment, with the goal of tailoring the drug more effectively to the specific needs of T2D patients.

## Genetics and pharmacogenomics for personalized metformin treatment

An essential aspect of personalized medicine in diabetes management is its ability to unveil the genetic factors influencing an individual’s predisposition to the condition. Genetic screening helps identify people at risk of diabetes, allowing for timely intervention with targeted preventive measures and lifestyle modifications (Petrie et al., 2018). Advancements in genetic testing has increased the knowledge of genetic aetiology of T2D, with the identification of nearly 80 susceptibility loci (Kleinberger and Pollin, 2015; Hara et al., 2014). Aside from genetic variations that may be causative factors in diabetes, individual patient attributes that influence how well a given medication works have major impact on personalized diabetes care.

From pharmacogenetics, pharmacogenomics is a method that has expanded into new areas for the pharmaceutical and biomedical industries (Khoury, 2003). Pharmacogenomics enhances personalized medication regiment according to a patient’s genetic and genomic characteristics. The field has the potential for a novel approach to drug selection by optimizing pharmacokinetics and pharmacodynamics to improve drug efficacy and safety (Sadée and Dai, 2005) by studying the gene expression over time (Bozkurt et al., 2007). Gene content or a person’s DNA content remains constant throughout time; however, the amount of RNA or how much a gene is utilized fluctuates. Determining the genes products across time adds further complexity to the process of identifying genes that are crucial to the disease (Klonoff, 2008).

Candidate genes and genome-wide association studies have determined genes with variations in sequence that are correlated with response (decrease or increase) to metformin in various study populations (Takane et al., 2008; Shikata et al., 2007; Becker et al., 2009a). As metformin is not metabolised but rather transported and excreted unchanged, studies have highlighted the role of genetic variations in genes associated with organic cation transporters (OCT) and multidrug and toxin extrusion (MATE) proteins in influencing metformin response. Genetic variations in SLC22A1 (OCT1) have been linked to altered metformin uptake and response. For example, individuals containing minor C allele in single nucleotide polymorphism (SNP) rs622342 of the gene SLC22A1 showed slower reduction in glucose after metformin therapy (Becker et al., 2009b; Naja et al., 2020).

Functional variants such as R61C, M420del, G465R, G401S were associated with poor response to metformin and AMPK activation, potentially affecting glucose levels and insulin response in individuals according to study conducted by Shu et al. (Shu et al., 2007). Another prospective study demonstrated that the same reduced functional variants of OCT1 (M420del, R61C, G465R, and G401S) were linked to trough concentrations of metformin and progress in HbA1c post 6 months of metformin treatment in a group of 151 diabetic patients in South Danish cohort (Christensen et al., 2011). On the contrary, M420del polymorphism of OCT1 is associated with good response to metformin in patients with newly diagnosed diabetic individuals. The study focused on responders and non-responders, where the former had significantly lower frequency of M420del mutant allele compared to the latter (Mahrooz et al., 2015). In another prospective observational study, researchers found that a specific genetic variation (rs72552763 SNP) in the SLC22A1 gene is associated with better metformin response. Individuals with the deletion version (del_G) had higher metformin response and lower HbA1 levels compared to those with the common GAT-GAT (G-G) genotype. While the association was not replicated at the allele level, the del_G was consistently linked to improved glycemic control (Degaga et al., 2023). In a Japanese cohort, 2 variants of SLC22A1, rs4646272 and rs628031, are found to have negative and positive associations with metformin efficacy, respectively (Shikata et al., 2007).

Empirically supported studies demonstrated that genetic polymorphisms of SLC22A2 (OCT2) cause inter-patient heterogeneity in the metformin uptake and clearance (Takane et al., 2008; Todd and Florez, 2014). Studies in Korean and Chinese populations indicated decreased metformin clearance and increased plasma concentrations in carriers of c.596C > T, c.602C > T, c.808G > T variants, implying the need for potential adjustments in dosage to optimize metformin treatment (Song et al., 2008). Multiple studies have failed to provide a connection between OCT2 variants and metformin response (Sundelin et al., 2017; Kerb et al., 2002; Rena et al., 2013; Dujic et al., 2017). However, it has been suggested that C allele of rs8192675 in the gene SLC2A2, which encodes GLUT2 (glucose transporter), is essential for regulating metformin action (Zhou et al., 2016). A recent meta-analysis studied 13 OCT2 variants with varying frequency and its impact of metformin efficacy in a mixed ethnic cohort, among which one variant (rs316019) displayed both positive and negative effects in pharmacokinetics and pharmacodynamics of the drug (Borra et al., 2023) (Peng et al., 2023).

Similarly, variations in the MATE1 gene (SLC47A1) have been associated with changes in metformin’s capability to alter glucose levels. Becker et al. have demonstrated that homozygous carriers of SLC47A1 rs2289669 A-allele show a larger reduction in HbA1c to metformin therapy compared to the prevalent G-allele (Becker et al., 2009a). Another study also showed that the diabetic patients with homozygous A-allele showed 2-fold decrease in HbA1c in contrast to patients carrying G-allele (Tkáč et al., 2013). Polymorphism rs622342 A > C was associated with lower reduction in HbA1c, hence, genetic variation at rs622342 is correlated with hypoglycemic effect of metformin in diabetes (Becker et al., 2009b). Although, meta-analysis of Metformin Genetics Consortium identified no significant association between metformin-related glycemic response and MATE1 transporter genes (Dujic et al., 2017). Therefore, findings are inconsistent for metformin response in this gene (Christensen et al., 2011; Dujic et al., 2017; Tkáč et al., 2013; Jablonski et al., 2010).

**FIGURE 2:**
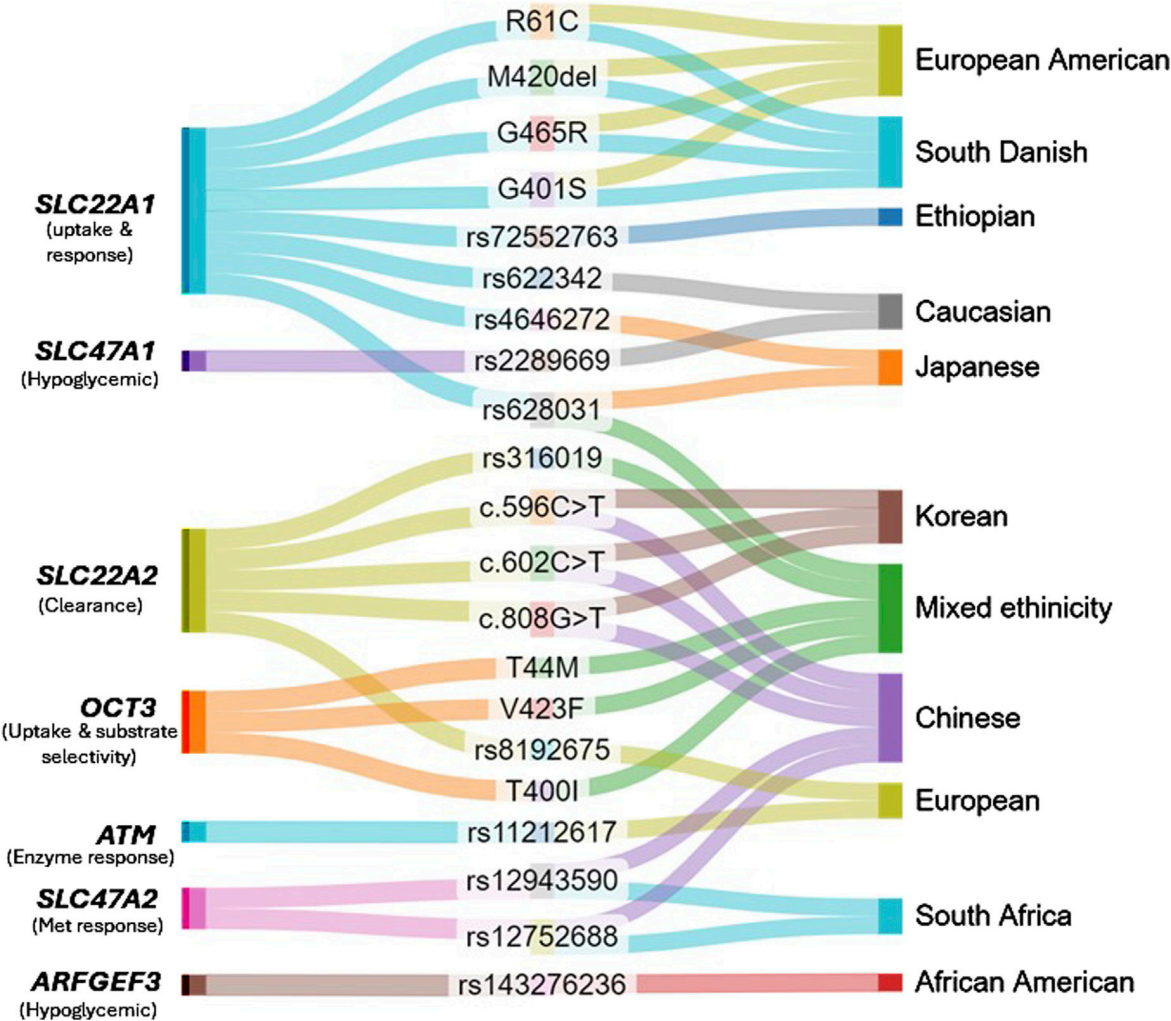
Variants associated with metformin action in T2D population.

Pharmacogenomics studies have identified additional genetic variations associated with changes in metformin response in diverse populations. A genome-wide association study demonstrated that the serine/threonine kinase encoding - ATM gene (ataxia telangiectasia mutated) has potential involvement in the mechanism of enzymes responsible for response to metformin (Zhou et al., 2011). According to another pharmacogenetics study, the metformin transporters OCT1, OCT2, OCT3, and P-GP are not associated with treatment inefficacy in Mexican type 2 diabetes patients (Hemauer et al., 2010). OCT3 variants T44M, V423F, and T400I were exhibiting decreased metformin uptake and changed substrate selectivity in another study by Chen et al. (Chen et al., 2010). In addition, a recent study has identified a novel variant (rs143276236) in the gene ARFGEF3 in the discovery cohort with a potential to reduce HbA1c in individuals with metformin monotherapy in African-Americans, although this find was not significant in the validation cohort (Wu et al., 2023). A correlation was found between the GA genotype of SLC47A2 rs12943590 and reduced response to metformin therapy, and CT genotype of rs12752688 was strongly associated with improved response to metformin therapy (Xhakaza et al., 2020). Evidently, continuous efforts are made to identify potential genetic causes that alter response to metformin therapy worldwide. Even with the identified transporter genes of metformin, the reaction to therapy is remarkably diverse across various ethnicities (Ochi et al., 2024). It is imperative that further studies are crucial to confirm these findings across larger and diverse ethnic populations. Current pharmacogenomics of metformin are depicted in Figure 2.

Although pharmacogenomics has advanced considerably in connecting drug responses to genetic variations, it does not account for the influence of environmental factors and the interplay between the metabolism of the individual and the gut microbiome. There’s growing evidence that alternative sites of activity, like the gut microbiome, tissue-resident immune cells, and the gastrointestinal system, may potentially be significant in metformin efficacy (Thakkar et al., 2013; Lalau et al., 2021). It has been reported that the variation in metformin response due to genetic variation is minimal and majority of the difference can be explained by environmental and lifestyle factors (Zhou et al., 2014). In this context, metabolomics provides an integrative technique to explore the variation of metabolites in response to alterations in physiological systems, which can offer a novel insight to the varied reactions to treatment.

## Metabolomics and metformin treatment

Metabolomics is a subset of omics tools that concentrates on the biological changes that closely reflect the endpoints of biochemical events. It includes extensive biological changes from the genomics to proteomics (Johnson and Gonzalez, 2012). Metabolomics is the study of small molecules, in a body fluid or tissue extract, involved in cellular processes. It provides a screenshot of the phenotype of an individual and evaluates the alterations caused by drug intake, thereby increasing the understanding of mechanism of drug action and heterogeneity observed in drug response (Shahisavandi et al., 2023; Bain et al., 2009). Pharmacometabolomics is a valuable method for examining the alterations in metabolites resulting from disorders or environmental factors and predicting the efficiency or potential toxicity of a specific drug intervention (Corona et al., 2012). It aids in the understanding of pharmacokinetics and pharmacogenetics, providing knowledge of the processes underlying medication responses at the level of genetics (Corona et al., 2012). Multiple studies have reported on the association between the progression of diabetes and alterations in metabolites after hypoglycemic treatments (Zhao et al., 2010; Cai et al., 2009).

It is important to note that metformin can significantly affect the levels of various metabolites, including those involved in the glucose metabolism, lipid metabolism, tricarboxylic acid cycle (TCA), and urea cycle (t Hart et al., 2018a). A comprehensive investigation concluded that, regardless of the metabolic status or disease condition, metformin administration significantly changed the metabolic profiles linked with multiple pathways (Kim, 2021b). For example, a study found that metformin-related metabolites, such as eicosanoids, hydroxyl-methyl uracil, propionic acid, and glycerol-phospholipids, significantly altered in those who took a single dose of metformin with no underlying medical issues (Dahabiyeh et al., 2021). Therefore, it is conceivable that the metabolic profiles of individuals using metformin may undergo alterations, leading to distinctions in the profiles of responders and non-responders to the drug. To determine the metabolic profiles of metformin users and identify those who respond well or poorly, metabolomics research is essential. In the case of metformin therapy, this strategy is crucial for developing precision medicine and customizing treatment plans. For example, a pharmacometabolomic approach has been proposed with the potential to identify distinct metabolic signatures associated with response to the metformin, paving the way for personalized metformin therapy strategies (Naja et al., 2023).

A study by Safai et al. demonstrated that metformin therapy is correlated with reduced amino acids, such as, tyrosine, valine, and carnitine in serum, which contributes to insulin resistance and mitochondrial dysfunction. Interestingly, this study found no metabolites that indicate or associate with HbA1c-lowering effect of metformin (Safai et al., 2018). Another study has reported that metformin seemingly increases the production of 3-hydroxy fatty acids (3-HFAs) either by beta-oxidation or via gut microbiota. 3-HFAs can activate G protein-coupled receptors and thereby regulate the pleiotropic effects of metformin (Naja et al., 2024). Additionally, alanine is reported to be significantly increased with metformin monotherapy and combined with sulfonylurea, and branched-chain amino acids (valine, leucine, and isoleucine) are increased with metformin therapy (t Hart et al., 2018b).

It is possible to define the mechanisms of heterogeneity in response to medication by combining the baseline data on metabotype with signature of drug exposure. Various studies have explored the impact of metformin on the plasma metabolome in diabetes. Gormsen et al. has reported that 1,000 mg of metformin twice a day improved the fasting plasma glucose level in only metformin-treated diabetes and the metabolite 1,5-anhydroglucitol (1,5-AG) was associated with glucose-lowering effect (Naja et al., 2023; Gormsen et al., 2019). A cross-sectional and longitudinal study using LC-MS and non-targeted metabolomics reported that citrulline showed lower relative concentrations in individuals with metformin-treated T2D individuals compared to those without metformin treatment in the human study. This effect was confirmed in mice, where significantly lower citrulline values were observed in plasma, skeletal muscle, and adipose tissue of metformin-treated animals (Adam et al., 2016). This result was also consistent with another study by Breier et al. where citrulline was reduced in serum after metformin treatment in a cohort of previously untreated diabetic patients (Breier et al., 2017). Irving et al. conducted a randomized, double-blind, placebo-controlled design, it was observed that the combination therapy of pioglitazone and metformin led to a reduction in the concentrations of citrulline and arginine (Irving et al., 2015). These studies suggest that metformin may influence downregulating the urea cycle in T2D. However, the metabolic signature associated with good and poor response to metformin therapy is crucial to adjust the treatment that best suit the patient. In line with this, only a handful of studies have investigated the differences in metabolites between responders and non-responders. Park et al. has reported three metabolites, hippuric acid, citric acid, and myoinositol as potential diagnostic biomarkers to predict metformin response (Park et al., 2018). Naja et al. has shown that sphingomyelins, acylcholines, and glutathione metabolites were increased in good responders (Naja et al., 2023). A recent review has summarised studies focusing on metabolic patterns related to metformin response in pre-diabetes and diabetes (Kim, 2021b). A concise table presenting the discussed studies are presented in Table 1.

**TABLE 1:** Summary of metabolites in relation to metformin treatment and its response.

Metabolite(s)	Observation	Associated mechanisms
Eicosanoids, glycerophospholipids, 5 hydroxyl-methyl uracil, Propionic acid	Inverse relationship with metformin levels	Reduce inflammation and dyslipidemia, alter DNA lesion repair mechanisms, and suppress appetite (Dahabiyeh et al., 2021)
Tyrosine, valine, carnitine	Reduced with metformin intake	No direct connection to lowering HbA1c levels (Safai et al., 2018)
Alanine	Significantly increased with metformin monotherapy and combined with sulfonylurea	Metformin-treatment associated metabolite (t Hart et al., 2018b)
3-hydroxy fatty acids (3-hydroxydecanoate, and 3-hydroxyoctanoate)	Increased in metformin therapy	Associated with mitochondrial β-oxidation (Naja et al., 2024)
Branched-chain amino acids (valine, leucine, and isoleucine)	Decrease?	Suppress BCAA activity and PI3K/Akt/mTOR pathway
1,5-anhydroglucitol (1,5-AG)	Reported in fasting plasma of metformin-treated diabetes patients	Glucose lowering effect in metformin users (Naja et al., 2023; Gormsen et al., 2019)
Citrulline	Lower concentration after metformin use	Observed in plasma, skeletal muscle, and adipose tissue in human and mouse studies. Indication of insulin sensitization (Adam et al., 2016)
Hippuric acid, Citric acid, Myoinositol	Lowered levels in the metformin responders	Proposed as diagnostic markers for metformin responders vs. non-responders (Park et al., 2018)
Sphingomyelins, Acylcholines, Glutathione	Increased levels in individuals showing response to metformin	Potential biomarkers of favourable metformin response (Naja et al., 2023)

Metabolomic research on metformin treatment began in the early 2010s and has grown since, however, the number of publications based on clinical research is limited. Studies have shown that metformin is correlated with TCA cycle, lipid metabolism, glucose metabolism, although the results were inconsistent between studies and not enough to derive robust conclusions. Moreover, the metabolite markers associated with responders and non-responders to metformin are not clearly established as either a cause or an effect of genetic alterations on the pharmacokinetics of metformin (Florez, 2017). However, these metabolites may be helpful in predicting how people would react to metformin and facilitate categorization of individuals based on their responses to the treatment, and thereby paving the way for individualized metformin treatment plans.

As previously discussed, metformin users have heterogeneous response to hyperglycemia in diabetes patients (Pearson, 2016). Metabolomics is, nonetheless, a powerful approach to discover the complex metabolic interactions and metabolic patterns in health and disease. The microbiome, medication, lifestyle, and other environmental factors have a significant impact on how differently individuals respond to different drugs. There is mounting evidence that the microbiota—more especially, the gut microbiota—plays a role in drug metabolism (Kim, 2021b; Vernocchi et al., 2016). The microbiota is responsible in multiple biochemical functions directly correlated with the host, investigation of metabolic interactions between the gut microbiota and host metabolism with provide a comprehensive view of the genetics-environment-health connection.

## Pharmacomicrobiomics

The gastrointestinal tract is home to a varied and dynamic colony of bacteria known as the gut microbiota, which has a major influence on human health and disease (Clemente et al., 2012). These microbes are necessary for immune system modulation, nutritional absorption and digestion, and the synthesis of critical metabolites (Thursby and Juge, 2017). A number of factors, including as medication use, lifestyle choices, and nutrition, affect the makeup and functionality of the gut microbiota (Hasan and Yang, 2019). In turn, gut metabolites can underline how lifestyle and dietary habits can influence the response to different treatments (Vernocchi et al., 2016).

### Gut diversity as a marker for metformin responsiveness

Microbes can influence drug pharmacokinetics through mechanisms such as activation, competition, biodegradation, and potentiation. Microbes can play a direct role in the activation and inactivation of drugs via biotransformation of drug molecules into secondary metabolites (Enright et al., 2016). They can indirectly influence the drug response by generating microbial metabolites that interfere with gene expression and host signaling pathways (Haiser et al., 2013). Moreover, modifications to the gut microbiome can result in variability in drug responses (Panebianco et al., 2018). Pharmacomicrobiomics is the study of synergistic effects between the drug and gut microbiome and explains the impact of variations in microbiome on drug action and response (Aziz et al., 2011; Saad et al., 2012). The complex relationship between the gut microbiota and metabolic health, especially in the context of T2D is crucial for understanding and treating the condition. The gut microbiome has an impact on the host metabolism, influencing insulin secretion and intestinal development and playing a significant role in metabolic disorders. Evidence from multiple microbiota transplantation studies suggest that modulation of microbiome could improve metabolic health of individuals. As diabetes is associated with alterations in the microbial profiles and reduced diversity, microbiome may contribute to the severity and progression of the disease (Vrieze et al., 2012; Wang et al., 2011; Koeth et al., 2013). A recent review has summarized the direct effects of gut microbiota on the mechanisms involved in the development of T2D, underlining the alterations on the microbial composition due to different therapeutic interventions, and its effect on insulin resistance and glycemic control (Barlow and Mathur, 2022). Taken together, these studies indicate potential therapeutic strategies for managing T2D by restoring or altering the gut microbial composition.

The study of the microbiome in connection to metformin response is gaining a lot of attention, with possible applications to personalized therapy. Developing microbiome-targeting methodologies by understanding the role of gut microbiome in response to drugs can improve drug efficiency and reduce adverse drug effects (Lee H. L. et al., 2018; Tsunoda et al., 2021). Utilizing microbiome modulators to treat metformin sensitivity has previously been reported to be successful according to the research by Burton et al. (Burton et al., 2015). Evidently, pharmacomicrobiomics is becoming a crucial aspect of personalized medicine and modifying the gut microbiome has the potential to manage drug efficiency and safety at the individual level. (Ting et al., 2022).

### Gut microbiota composition changes due to metformin

Metformin is increasingly recognised for its role in the gut, including modifications to the microbiome which might account for some of the drug’s therapeutic efficacy and side effects (McCreight et al., 2016; Forslund et al., 2015). In addition to many functions such as, enhancing the absorption of glucose, the formation of glycolytic lactate, the secretion of glucagon-like peptide-1 (GLP-1), and the bile acid pool, metformin alters the microbiome composition in the gut (Wu T. et al., 2017). For example, a study by Lee et al. (Lee Y. et al., 2021) demonstrated that the administration of metformin changed the relative abundances of metabolites such as carbohydrates, amino acids, and fatty acids as well as specific gut bacteria (*Escherichia, Romboutsia, Intestinibacter,* and *Clostridium*). These alterations were linked to important metabolic processes that are implicated in the hypoglycemic effect of metformin via AMPK activation, including gluconeogenesis, energy metabolism, and branched-chain amino acid metabolism (Lee Y. et al., 2021; Wang et al., 2022; Damanhouri et al., 2023).

Notably, the gut serves as the main human reservoir for orally ingested metformin with 100–300 times higher than in serum (Wilcock and Bailey, 1994; Foretz et al., 2023b). In fact, it is apparent that the microbiome is the main source of metabolic interactions (Wu H. et al., 2017; Shin N. R. et al., 2014). The microbiome is altered in response to genetic influences (Goodrich et al., 2014), environmental factors (Nagpal et al., 2017), and lifestyle (Lee S. H. et al., 2018), and taken together these factors create a unique microbial signature of a person. Additionally, not everyone benefits equally from metformin therapy, some experience negative effects as well (DeFronzo and Goodman, 1995) and therefore the microbiome is an important part in the inter-individual variability in drug response. These facts present intriguing concerns regarding the molecular mechanisms by which metformin affects the gut microbiota and involvement of the gut microbiota with respect to tolerance or intolerance to metformin. Metformin-treated diabetic patients have shown evidence of gut dysbiosis, however, only hypothetical mechanisms of metformin affecting glucose regulation in the gut exist so far (Buse et al., 2016). The causal relationship reported between the unique microbial metabolic pathways found in distinct gut microbiota compositions and the long-term durability of the glycemic response to metformin monotherapy in T2D further emphasised on the interplay between metformin and gut microbiota metabolism (Foretz et al., 2023a). For example, thiamine biosynthesis pathways may have a role in maintaining the glycemic response to metformin monotherapy over time. (Hung et al., 2021).

Investigations conducted in animal models validated that metformin-induced modulation of gut microbiome resulted in the formation of short-chain fatty acids (SCFAs), reduced lipopolysaccharides in circulation, impeded intestinal proinflammatory signalling and altered hepatic uptake of metformin (Ahmadi et al., 2020; Zhang et al., 2019; Liu et al., 2021; Wu et al., 2019). This is particularly interesting as SCFA-producing bacteria such as *Butyrivibrio* and *Roseburia* ferment indigestible complex carbohydrates and enhance insulin sensitivity (Pedersen et al., 2016). Forslund et al. proposed that the microbial production of SCFAs mediates the efficacy of metformin in the treatment of type 2 diabetes by enhancing glucose regulation.

Several mechanisms have been hypothesized linking metformin action on the GM and the improvement of glucose tolerance (Foretz et al., 2023a). In a study involving *in vivo* models, altered hepatic uptake of metformin was observed in pseudo-germ-free rats compared to conventional diabetic rats. The pharmacodynamic and pharmacokinetic property of metformin was modified in the pseudo-germ-free rats, possibly due to decreased OCT1 expression (Wu et al., 2019). Another study showed that bile acid glycoursodeoxycholic acid (GUDCA) was increased and *Bacteroides fragilis* was decreased in newly diagnosed T2D patients under metformin treatment. Additionally, the benefits of metformin were eliminated in high fat diet (HFD)-fed mice colonized with *B. fragilis*, suggesting that the intestinal farnesoid X receptor (FXR) axis mediated the glucose-lowering effect of metformin (Sun et al., 2018). In contrast, Silamikele at el. has reported an elevated abundance in *B. fragilis* in response to metformin treatment in HFD groups of both sexes of mouse model (Silamiķele et al., 2021). Hence, further investigations are required to elucidate the exact effect of *B. fragilis* on metformin efficacy.

### Role of gut microbiota in modulating metformin efficacy

Studies have reported the influence of gut microbiota community on the hypoglycemic effect of metformin in T2D individuals. An *in silico* human microbiota metabolic modeling study providing evidence for metformin–microbiota interactions revealed a projected increase in agmatine synthesis capability by *E. coli* in metformin-treated T2D patients (Pryor et al., 2019). A randomised clinical trial demonstrated that metformin improved hyperglycemia in T2D by increasing *Blautia* and *Faecalibacterium* in the gut (Tong et al., 2018). In another double-blind randomized study, metformin altered the gut microbiome by increasing *Escherichia* and *Akkermansia muciniphila* in individuals with treatment naïve T2D. This study examined the metabolic benefits of metformin by transplanting the fecal samples from donors who were treated with metformin to germ-free mice. It was speculated that glucose tolerance was enhanced either by elevating the production of SCFAs or modifying plasma bile acid composition (Wu H. et al., 2017). Based on these data, it is understood that metformin’s ability to treat type 2 diabetes may be linked to its impact on the gut microbiota and its generation of short-chain fatty acids (SCFAs). In a recent systematic review, the species *A. muciniphila* and many taxa in the order *Enterobacteriales* were found to be more prevalent in insulin resistant (pre-diabetic) and T2D patients using metformin (Cao et al., 2020). The underlying mechanism is not fully debunked, although, *A. muciniphila* is found in the mucus layer of the colon responsible for secretion of mucus and improving the integrity of the intestine by decreasing the permeability of the epithelial layer (Geerlings et al., 2018). Metformin is also correlated with an improvement in the thickness of mucin-secreting cells (Induri et al., 2022). Interestingly, in a mouse model with impeded bile acid flow, FXR axis was activated by ligand TC-100, which prevents the intestinal mucosal damage, and showed association with increase in *A. muciniphila* (Marzano et al., 2022). This study emphasizes on the preservation of intestinal barrier which plays a metabolic role in regulating inflammation in the intestines, which could eventually lead to management of adipose tissue dysfunction, chronic systemic inflammation, and glucose intolerance in T2D. Li et al. discovered that *A. muciniphila* enhances HFD-induced intestinal hyperpermeability and influences the intestinal barrier function via upregulation of tight junction (TJ) closure proteins (Li et al., 2016), thereby reducing the circulation of pro-inflammatory lipopolysaccharides (LPS) and inflammation. In line with the research that showed increase in the abundance of *Akkermansia* species, it has been demonstrated that use of metformin was essentially associated with an increased production of SCFAs (Wu H. et al., 2017; Ejtahed et al., 2019; Huang et al., 2018).

Additionally, metformin treatment shows higher abundance of *Bifidobacterium* which can stabilize the GI mucosa by increasing the production of gastric mucin. Increasing the amount of *Bifidobacterium* in the gut led to a larger level of proglucagon mRNA, which in turn encouraged the production of glucagon-derived peptides (Cani et al., 2009). In the process, glucagon-like peptide-2 (GLP-2) increased the proliferation of intestinal epithelial cells and decreased permeability, thus strengthening the intestinal mucosal barrier. Together with the ability of metformin to improve intestinal barrier integrity and strengthen it by increasing intestinal expression of TJ proteins, these data suggest that metformin also indirectly reduces insulin resistance through modulating GM composition, SCFA levels, and intestinal barrier integrity.

### Bidirectional relationship between metformin and gut microbiota

It is important to note that the relationship between metformin and the gut microbiota is bidirectional. Metformin affects the gut microbiota composition, which in turn may influence its therapeutic effects. Study by Ezzamouri et al. used metabolic modeling to understand the metabolic interactions between the human gut microbiome and metformin treatment in diabetes patients. The research provided insight into the metabolic contribution of the gut microbiota and the effect of metformin treatment, including the changes in the abundance of specific microbial species (increased *Blautia wexlerae,* a short chain fatty acid-producing species) and decreased *Alistipes obesi* (Alistipes genera), *Roseburia* sp*. CAG:100, Faecalibacterium prausnitzii 7, F. prausnitzii 3 (L2–6),* butyrate producers, and several firmicutes bacteria) in response to metformin (Ezzamouri et al., 2023). In line with this context, the metabolites generated by the gut microflora can affect how well metformin works as a treatment contributing to interindividual variability in the drug’s response. For instance, metabolomic analysis of plasma from metformin-treated individuals with high blood glucose levels and those with low blood glucose levels revealed a microbial metabolite, imidazole propionate, as a negative regulator of metformin efficacy in humans (Koh et al., 2020). Imidazole propionate lessened the immediate glucose-lowering impact of metformin in mice fed a western diet by inhibiting AMPK signaling in the liver via p38γ-dependent mechanism. It's interesting to note that p38γ inhibition blocked imidazole propionate’s inhibitory effect on acute metformin action, suggesting possible therapy options for T2D patients who do not respond well to metformin (Koh et al., 2020).

Several studies have demonstrated that metformin can alter the composition of the gut microbiota in both healthy individuals (Ejtahed et al., 2019; Bryrup et al., 2019) and in diabetic patients (Forslund et al., 2015; Wu H. et al., 2017; Sun et al., 2018; Tong et al., 2018), however, studies have not fully investigated the difference in microbiota composition between responders and non-responders under metformin therapy. This is an essential point to consider when assessing the inter-variability in the response to metformin, as it helps to understand the gut microbiota’s contribution to the unresponsiveness.

Notably, metformin also causes unfavourable side effects, for example, increased relative abundance of *Escherichia spp*. and its virulence factors, causing bloating, diarrhea, and vomiting (Forslund et al., 2015) in some individuals. Increase in Enterobacteriaceae species, such as *Salmonella*, *Klebsiella*, *Shigella*, and *Escherichia*, was observed in independent cohorts of European women (Karlsson et al., 2013) and Nordic (Huang et al., 2018) diabetic patients taking metformin. In another cohort from Colombia, metformin administration significantly boosted *Prevotella* and *Megasphaera* genus, responsible for bacterial vaginosis, while reducing Clostridiaceae 02d06, *Oscillospira* and *Barnesiellaceae* (de la Cuesta-Zuluaga et al., 2017). Individuals under metformin treatment showed positive correlations were found with *Bacteroids* and *Escherichia* species and negative associations were obsevered with *Ruminocococus* and *Faecalibacterium* even with shorter duration of metformin treatment in Japanese cohort (Li et al., 2021). In short, in some cohorts metformin treatment essentially alters the gut microbiota in a way the “good” bacteria are reduced and the virulent or pathogenic species are increased.

Alpha diversity is a measure of species diversity in an ecosystem, and Shannon diversity is a metric of alpha diversity commonly used in gut microbiome studies as a marker for metabolic health (Yin et al., 2019; Kim et al., 2017). Wilmanski et al. have shown that 1-carboxyphenylalanine and methyl glucopyranoside (α+β) were among the 11 strong predictors of Shannon α-diversity of gut microbiota (Wilmanski et al., 2019). 1-carboxyphenyalanine is reported to be associated with poor response to metformin (Naja et al., 2023) and with lower microbiome diversity (Wilmanski et al., 2019), and previously shown to be most discriminating metabolite of insulin resistance (Almuraikhy et al., 2023; Diboun et al., 2021). In this context, patients under metformin and probiotics had a larger reduction in glycated hemoglobin levels compared to those under metformin-monotherapy (Şahin et al., 2022). As discussed earlier, metformin affects relative abundance of various phyla that may lead to gastrointestinal issues independent of potential influence on glycemic control which can lead to intolerance to metformin (Forslund et al., 2015; Adeshirlarijaney and Gewirtz, 2020). Up to 30% of individuals on metformin are considered to experience undesirable gastrointestinal side effects (Forslund et al., 2015). The impact of metformin on gut microbiota is known for both its beneficial effects and unfavourable gastrointestinal side effects, which can result in suspension or drastically lowering the daily dosages, and thereby reducing the medication’s effectiveness as a first-line antidiabetic treatment. In order to limit these detrimental effects, probiotic treatment, which alters the microbiota, has also been demonstrated to improve insulin resistance by boosting the abundance of SCFA producing bacteria and modulating bile acid metabolism, possibly improving glucose regulation while working alongside metformin (Shin N. R. et al., 2014; Şahin et al., 2022; Chen et al., 2023). Probiotics and prebiotics alter the gut microbiota and enhance the effects of metformin therapy on T2D in both human and animal studies (Foretz et al., 2023a; Seicaru et al., 2022).

It is challenging to draw robust conclusions because of the wide range of roles played by the microbiome, the variety of microorganisms that are there, and the numerous variables that can affect their activity. A systems-based approach and customized drug testing techniques may be required to gain a deeper understanding of the underlying mechanisms and causes (Aziz et al., 2011). This may contribute to the explanation of the distinct variables that may impact drug-microbe-host interactions and offer more individualized therapeutic options. Therefore, further research is necessary to clarify the many functions that various bacteria play in host metabolism.

## Integration of pharmacogenomics, metabolomics and microbiomics: Path to personalized metformin therapy

**FIGURE 3:**
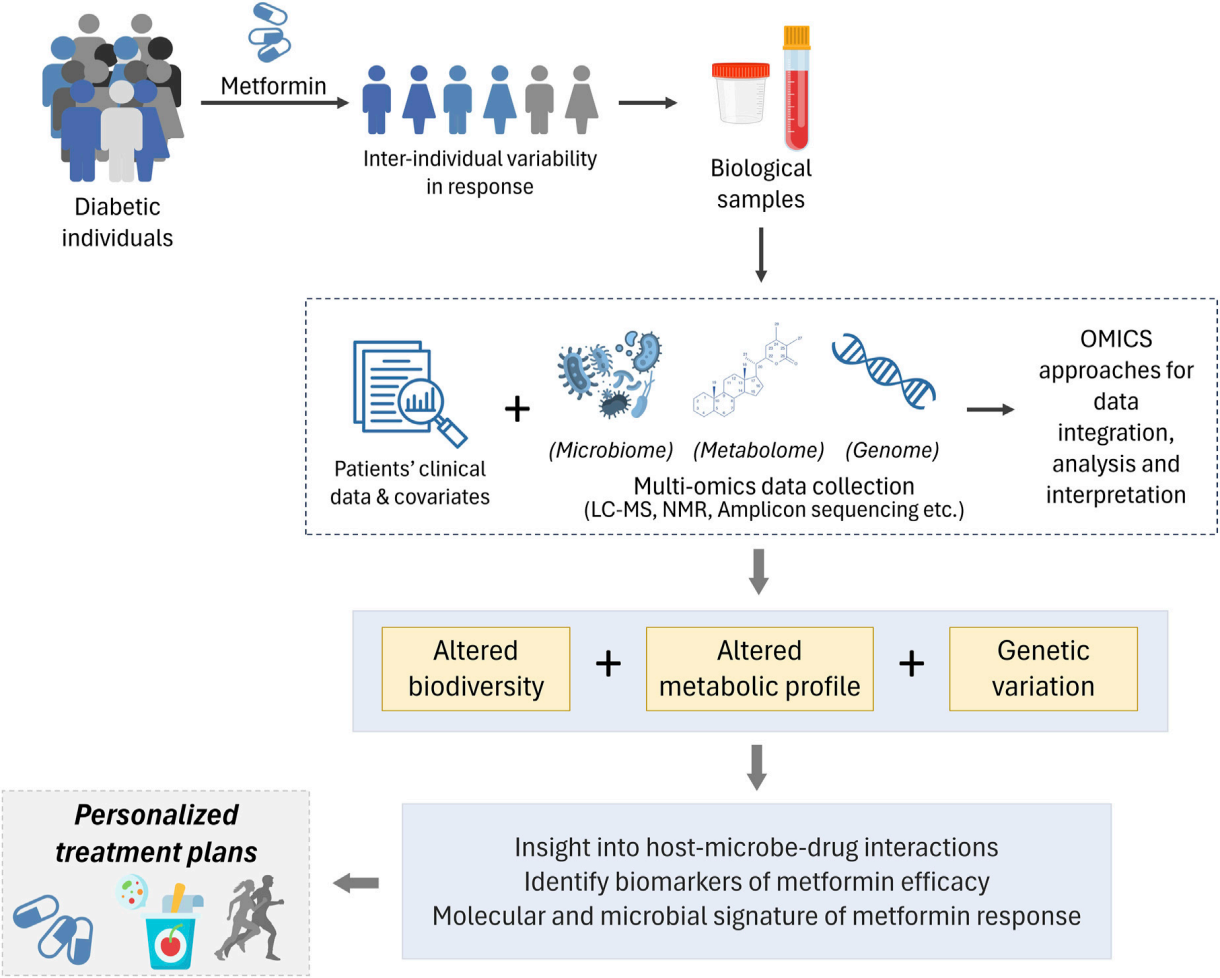
Inter-individual variability in response to metformin therapy is observed T2D individuals. Biological samples (such as blood, faeces, etc.) from a well-defined cohort is used to derive patient information. Followed by leveraging the power of multi-omics to integrate clinical parametric data, along with genomics, metabolomics and microbiomics to decipher inherent genetic variations, altered biodiversity and metabolic profile to further our understanding of diverse responses to metformin therapy and enabling personalized treatment options.

Until recently, personalized medicine in T2D has predominantly focused on variation in DNA sequence; however, this captures only a part of the overall complexity of individual variation. The genotype of an individual does not explain the dynamic process impacted by several environmental and/or disease-related factors, however, the metabotype serves as the culmination of the “genome-environment-microbiome” interplay (Bafiti and Katsila, 2022; Beger et al., 2016). Personalized medicine is pacing towards combining clinical and molecular signatures that can forecast treatment outcomes and decrease the ambiguity in deciding suitable treatment. Clinical care of diabetes patients emphasizes on reducing plasma glucose levels and the risk of complications associated with the condition. However, significant variability is present in response to the same intervention. To carry out the most appropriate intervention plan based on a patient’s particular characteristics, a deeper understanding of the underlying reasons of various pharmacological reactions is required. For this reason, studies that collect molecular signatures in terms of genes, metabolites and microbiomes can truly elucidate the complex interplay at hand (Figure 3).

Multi-omics interpretation of omics data is crucial for a thorough understanding of T2D and for their prognosis, diagnosis, and treatment. Advancements in technologies have provided exceptional opportunities to evaluate and combine individual omics data, which has captured biological variation to enable targeted therapeutic options. Walford et al., combined genetics, metabolomics and clinical parameters from 1,622 non-diabetic individuals enhanced the prediction of possible T2D diagnosis in the future. A 62-variant GRS showed an AUC of 64%, which increased to 88% with the combination of genetics, metabolomics, and clinical factors (Walford et al., 2014).

Since the early 2010s, studies have shown that integration of multiple data can enhance personalized therapeutic options in the field of diabetes research. For example, researchers have complied genomics, metabolomics, proteomics, and microbiome analyses in a single framework to develop individualized dietary interventions for T2D (Price et al., 2017). Genome-wide association studies (GWAS) coupled with metabolomic profiling has provided novel insights the role of inherited variation in blood metabolic diversity and potential opportunities for novel drug development in T2D (Shin S. Y. et al., 2014). Integration of data on dietary intake, physical activity, sleep, anthropometric measurements, and gut microbiota using machine learning algorithm predicted postprandial glycemic response to real-life meals, this enables personalized diets to reduce postprandial blood glucose (Zeevi et al., 2015). However, only a handful of studies exist to date that are specific to metformin therapy and inter-individual variability and are mentioned here. Combining metagenomics data with genome-scale metabolic modelling predicted the changes in the gut microbial species in response to metformin treatment, establishing guidelines on how dietary modifications can improve drug efficacy (Ezzamouri et al., 2023). Xu et al. combined both metabolomics and genomics data and identified metformin-associated metabolites (PC ae C36:4, PC ae C38:5, and PC ae C38:6), which are involved in the AMPK pathway associated with *FADS1* and *FADS2* genes (Xu et al., 2015). Furthermore, artificial intelligence has been useful in identifying complex patterns in multi-omics datasets. Deep learning methods have proven helpful in the development of biological mechanism prediction and disease prediction models in several conditions (Wang et al., 2023; Bokulich et al., 2022). For example, multi-omics variational encoders (MOVE), a deep learning framework identified novel associations between gut microbiota and metformin in T2D patients (Allesøe et al., 2023). Following the examples, it is indeed necessary to conduct integrated omics research to further our understanding on variability in the response to metformin therapy in diabetic individuals.

Major upstream causal factor is the genome, with metabolome, microbiome and other omics being mediators which are impacted by age, gender, demographic characteristics, and pharmacological intervention (Yu et al., 2024). Moreover, current literature suggests that gut microbiome is a strong contender in metformin therapy and the microbiome-regulated mechanisms involving alterations in the gut microbiota composition influence its pleiotropic effects (Hung et al., 2021; Pryor et al., 2019; Seicaru et al., 2022; Zhao et al., 2023). The integration of various omics can shed light on the interactions and relationships between various molecular entities and biological processes (Ivanisevic and Sewduth, 2023). In essence, when these fields are applied to well-categorized cohort, they can provide deeper understanding on metformin efficacy and the microflora contribution to the genetic-influenced drug metabolism and thereby improve the accuracy of individualized treatment plans.

Integration of omics involves collection of data from various sources, i.e., biological structure (cell, organ, tissue), samples (urine, blood), conditions (time-points, experimental groups) etc. Omics data is generated by different analytical techniques (DNA sequencing, mass spectrometry or nuclear magnetic resonance spectroscopy). Microbiomics is analysed using nucleotide sequencing, amplicon sequencing, metagenomics, and metatranscriptomics (Joshi et al., 2023). Depending on the interest or experimental design, the quality and quantity of omics data can defer largely. Following collection, multi-omics studies integrate omics data from various sources, keeping the biological information from each omics intact (Ivanisevic and Sewduth, 2023). The complexity of the field and the multi-disciplinary nature requires collaboration between researchers with knowledge of human biology, genetics and epidemiology, health professionals, data scientists, and bioinformaticians to address the issues and challenges associated with the integration (Yu et al., 2024).

Despite the ongoing research, integration of multi-omics is still in its early stages. Full implementation of integration for precision medicine in clinical practice requires further efforts. Firstly, robust and reproducible omics data is scarce. Even state-of-the-art omics technologies have not captured enough biological variation to allow the creation of logical and distinct categories, and to enable targeted therapy (Hu and Jia, 2021).

Integration of genomics with metabolomics have been previously performed in many studies (Xu et al., 2015; Inouye et al., 2010; Yousri et al., 2023), however, the integration of the microbiome with the other omics is challenging and still in its infancy (Mallick et al., 2017). Microbiome data is sparse where several taxa are detectable in only a few samples and therefore require robust statistical methods. Microbiome and metabolome are measured as relative abundances, and this compositional nature is susceptible to bias and may result in misleading associations if not corrected. Adding control features with known concentrations to samples before profiling, data transformation methods like centered log-transformation, and data normalization processes, including more recently created methods like empirical Bayes normalization, are some strategies for resolving compositionality (Quinn et al., 2019; Kumar et al., 2018). It can also be challenging to interpret the results because many identified metabolites have unknown chemical identities. Additionally, ambiguity in the origin of known metabolites, that is, we are exactly not sure if the metabolite was consumed, made by the host, the outcome of a microbial metabolism, or the consequence of combination of these. Hence, human microbial reference genome databases and more extensive metabolite databases will be crucial for upcoming integrative analytic projects (Pasolli et al., 2019; Lee-Sarwar et al., 2020; Li et al., 2014). The diverse and complex nature of multi-omics data poses significant computational and statistical challenges in terms of data heterogeneity, integration, analysis, and validation (Vahabi and Michailidis, 2022). Moreover, ensuring data reliability and consistency across studies remains challenging.

To handle complexity of the microbiome reliably, additional investigation and advancement are required to enhance the multi-omics integration approaches and applications. The effort of integrating data for multi-omics investigations is complex and demanding, which necessitates rigorous planning and execution of the experimental design, including data acquisition, processing, and analysis. In addition, biological interpretation and validation of results requires comprehensive understanding of the relevant field and further experimental confirmation (Ivanisevic and Sewduth, 2023). For example, confirming the microbial origin of a metabolite requires *in vitro* culturing of microorganisms isolated from the faeces followed by looking for the desired compounds in the appropriate microbial extracts (Puig-Castellví et al., 2023).

The development and utilization of suitable analytical techniques capable of integrating multi-omics securely and reliably is further necessitated. Certain approaches to data interpretation are covered in recent research, particularly when handling big datasets such as predictive models (Subramanian et al., 2020), correlation-based analysis (Chen and Zhang, 2016), matrix factorization (Zhang et al., 2012), multiple kernel learning, and multi-step analysis (Ritchie et al., 2015). Researchers have reviewed and compiled several methods of integration and analysis of multi-omics data. A recent survey covering the statistical methodology for microbiome data analysis can be found here (Lutz et al., 2022). A guide to collect and combine multi-omics data was recently published by Athieniti and Spyrou in (Athieniti and Spyrou, 2023) and a comprehensive review of integrative methods and associated statistical methodologies by Vahabi and Michailidis can be found here (Vahabi and Michailidis, 2022).

Integrated approaches offer a way to use the integrated relationships seen in multi-omics data to better comprehend the inter-variability observed in metformin response, shedding a light on the microbial contribution to the drug efficiency (Ivanisevic and Sewduth, 2023). Our knowledge of how the gut microbiome affects the efficacy of metformin could further be improved by AI-based big data comparative studies of the gut microbiota with diverse population with drug usage. AI could help in finding and selecting a specific gut bacterial genera that can enhance drug efficacy (Huang et al., 2023).

## Conclusion

Integration of pharmacomicrobiomics to pharmacogenomics and pharmacometabolomics represents a significant stride towards personalized treatment of metformin in T2D. This comprehensive approach underlines the importance of moving beyond the traditional focus on genetic variations to incorporate the relationship between genetics, environment, lifestyle choices and their associated microbiome influence on individual’s health and disease outcomes (Li and Jia, 2013; Wilson and Nicholson, 2017; Lee C. B. et al., 2021). Through multi-omics, we can understand the mechanisms underlying the variability in drug responses which enables the tailoring of treatment options to the unique molecular and clinical signatures of each diabetic patient (Pearson, 2016).

However, the approach to integrating multi-omics into clinical practice is filled with challenges (Ivanisevic and Sewduth, 2023), such as the need for robust and reproducible data, the complexity of integrating various data types, and the computational and statistical difficulties involved in analysing the data. Despite the issues, advancements in analytical technologies, bioinformatics, and artificial intelligence are paving the way for more effective integration strategies (Huang et al., 2023). Ongoing research and collaboration across disciplines are essential to further our understanding of the multi-omics in addition to the significant progress that has already been made. As we continue to decipher the involvement of the genome, metabolome, and microbiome interactions in predicting the efficacy of metformin, we move closer to delivering personalized treatment strategies.
